# The Impact of Postpartum Posttraumatic Stress and Depression Symptoms on Couples’ Relationship Satisfaction: A Population-Based Prospective Study

**DOI:** 10.3389/fpsyg.2018.01728

**Published:** 2018-09-19

**Authors:** Susan Garthus-Niegel, Antje Horsch, Eric Handtke, Tilmann von Soest, Susan Ayers, Kerstin Weidner, Malin Eberhard-Gran

**Affiliations:** ^1^Department of Psychotherapy and Psychosomatic Medicine, Faculty of Medicine of the Technische Universität, Dresden, Germany; ^2^Department of Child Health, Norwegian Institute of Public Health, Nydalen, Norway; ^3^Institute of Higher Education and Research in Healthcare (IUFRS), Lausanne University Hospital, University of Lausanne, Lausanne, Switzerland; ^4^Department Woman-Mother-Child, Lausanne University Hospital, Lausanne, Switzerland; ^5^Department of Psychology, University of Oslo, Oslo, Norway; ^6^Centre for Maternal and Child Health Research, School of Health Sciences, City, University of London, London, United Kingdom; ^7^Health Services Research Unit, Akershus University Hospital, Lørenskog, Norway; ^8^Institute of Clinical Medicine Campus Ahus, University of Oslo, Oslo, Norway

**Keywords:** postpartum PTSD, couples, relationship satisfaction, depression, anxiety, Akershus Birth Cohort

## Abstract

The couple relationship is of particular importance in the transition to parenthood and in the early childhood years because it is related to the well-being and mental health of partners, children, and the family. One factor that may substantially influence relationship quality and couple satisfaction after childbirth is the woman’s experience of birth. Approximately 2–4% of women develop posttraumatic stress disorder (PTSD) after childbirth, with potentially wide-ranging negative consequences for the women themselves and their families. To date, some qualitative studies have explored the influence of postpartum PTSD on couple relationship satisfaction. However, quantitative studies are sparse, with mixed results and methodological limitations. We hypothesized that postpartum PTSD will be prospectively associated with low couple relationship satisfaction, even when taking into account a variety of potential confounding variables, and that the effect of postpartum PTSD symptoms on couple relationship satisfaction will be mediated by postpartum depression symptoms. This study is based on data from the Akershus Birth Cohort study, a prospective cohort study. Information from hospital records and questionnaires completed at 17 weeks gestational age, as well as at 8 weeks and 2 years postpartum were used (*n* = 1480). PTSD symptoms were measured by the Impact of Event Scale and couple relationship satisfaction was assessed using a modified version of the Mehrabians Marital Satisfaction Scale. Depressive symptoms were assessed by the Edinburgh Postnatal Depression Scale. Data were analyzed using bivariate correlations, multivariate regression analyses, and mediation analyses. Postpartum PTSD symptoms were prospectively related to low couple relationship satisfaction at 2 years postpartum, even when controlling for a considerable number of background factors. When including postpartum depression symptoms as predictor in the analyses, the effect of postpartum PTSD was no longer significant. Moreover, more detailed analyses showed that postpartum depression symptoms acted as a significant mediator, fully explaining the association of postpartum PTSD with couples’ relationship satisfaction. Early detection of couples’ relationship problems and the provision of professional help, particularly in high-risk couples may not only improve the quality of the couple relationship but also improve parenting and promote positive child outcomes.

## Introduction

Having satisfying long-term romantic relationships has been identified as one of the most important individual life goals (Roberts and Robins, 2000). Having a good relationship is associated with a range of positive personal outcomes, including better health and well-being, whereas relationship discord is associated with greater risk of psychopathology (Whisman, 2013). The couple relationship is of particular importance in the transition to parenthood and in the early childhood years because it is related to the well-being and mental health of partners, children, and the family (Redshaw and Martin, 2014). However, the transition to parenthood is associated with many psychological, social, and biological changes for women and their partners that may compromise relationship satisfaction (Horsch and Ayers, 2016). In addition, this period may also be challenging as a function of the baby’s health and sleeping patterns (Horsch and Ayers, 2016). Parents can feel overwhelmed by these changes and feel unable to cope with the new demands and responsibilities. Thus, many parents experience parenting stress. As a result, many couples may perceive a mismatch between their expectations and the realities of care in the postpartum period, combined with a lack of sleep and less opportunities to spend time together (Mitnick et al., 2009). In accordance with this notion, studies have shown a reduction in positive interactions, an increase in couple conflict, and a decline in couple relationship satisfaction after the birth of the first child (Doss et al., 2009; Mitnick et al., 2009; Kluwer, 2010).

One factor that may substantially influence relationship quality and couple satisfaction after childbirth is the woman’s experience of birth. Such experiences may have a long lasting impact on couple relationships, particularly if the birth and circumstances related to the birth are experienced as traumatic by the woman. The present study’s main aim is to examine this issue, and to examine mechanisms through which posttraumatic stress symptoms related to childbirth are prospectively associated with couple satisfaction.

In some cases, childbirth can involve exposure to actual or threatened death or severe injury of the woman and/or her child, thus meeting diagnostic criteria for a traumatic stressor (American Psychiatric Association, 2013). As a consequence, women giving birth can develop posttraumatic stress disorder (PTSD), which is characterized by re-experiencing, avoidance, numbing, and hyperarousal symptoms as well as negative cognitions and mood (American Psychiatric Association, 2013). Prevalence rates of postpartum PTSD have been estimated to range from 1 to 6% in community samples in Europe (Pantlen and Rohde, 2001; Ayers, 2004), the US (Soet et al., 2003), Australia (Creedy et al., 2000), Israel (Polachek et al., 2012), and Nigeria (Adewuya et al., 2006). The somewhat varying prevalence rates possibly reflect different culturally dependent social norms and expectations about childbirth. However, they may also reflect the differences in the provision of maternity care (Halperin et al., 2015).

A recent meta-analysis identified various risk factors for PTSD following childbirth: negative subjective birth experiences, having an operative birth, lack of support, and dissociation (Ayers et al., 2016). It also confirmed a strong co-morbidity with postpartum depression (Ayers et al., 2016). Moreover, approximately one in seven mothers may develop postpartum depression within the first 12 months following childbirth, characterized by feelings of low mood, loss of interest in usual activities, feelings of worthlessness, and loss of energy (Wisner et al., 2006). The prevalence of postpartum depression differs across countries from approximately 10% in high income countries to approximately 20% in low income countries (Gavin et al., 2005; Fisher et al., 2012; Gelaye et al., 2016). In developing countries, where mental health may be associated with even greater stigma and is often not covered by health insurance programs, postpartum depression is frequently not detected and appropriate treatment not offered (Sawyer et al., 2010; Gearing et al., 2015). Antenatal depression and anxiety, a history of depression, and a low level of partner support are the strongest independent predictors of postpartum depression (Milgrom et al., 2008).

Research shows that PTSD undermines positive relationship processes and/or exacerbates negative relationship processes between partners, including detrimental communication in the family and fear of intimacy, which in turn lowers the perception of the couple relationship quality (Campbell and Renshaw, 2013).

Focusing specifically on postpartum PTSD, some qualitative studies have examined its impact on the couple relationship. For instance, a recent systematic review and meta-synthesis of seven qualitative studies showed that childbirth-related PTSD can have a perceived negative impact on the couple relationship (Delicate et al., 2017). Five themes were identified: negative emotions, lack of understanding and support, loss of intimacy, strain on the relationship, and strengthened relationships. Another meta-synthesis of qualitative studies of traumatic childbirth included “shattered relationships” as a central theme, reflecting the fractious and difficult relationships that women who experienced a traumatic childbirth described with their infants and partners (Fenech and Thomson, 2014). This study found that most women characterized their relationships as negatively affected by the traumatic childbirth, resulting in long-term intimacy and sexual problems. Those sexual problems often led to relationships being strained. Also, some women felt they were no longer worthy of the relationship (Fenech and Thomson, 2014).

To date, quantitative studies examining the influence of postpartum PTSD on satisfaction with the couple relationship are sparse. One study with 64 couples who completed questionnaires 9 weeks after childbirth found no cross-sectional association between PTSD symptoms and couple relationship satisfaction (Ayers et al., 2007). However, this study was limited by a low response rate, a small sized sample, and a short follow-up period (9 weeks postpartum). Therefore, well-designed large-scale longitudinal studies examining the association between postpartum PTSD symptoms and subsequent couple relationship satisfaction are needed. Moreover, given such associations are found, studies examining potential mechanisms through which PTSD symptoms may influence couple relationship satisfaction are warranted. Postpartum depression may play a role in such potential mechanisms, as postpartum PTSD may lead to increased depressive symptoms in the woman. Such symptoms, including lack of energy, disinterest in social life, and irritability may in turn reduce couple relationship satisfaction (Zelkowitz and Milet, 1996; Wenzel et al., 2005; Hershkowitz et al., 2017). Similarly, anxiety in the postpartum period may arise as a result of symptoms of postpartum PTSD such as intrusion and hyperarousal, thereby leading to reduced couple relationship quality (McKenzie-McHarg et al., 2015; Agius et al., 2016). So far, research has only provided preliminary support for such potential mechanisms where maternal mental health problems mediate the association between postpartum PTSD symptoms and couple relationship satisfaction. More specifically, by examining the effect of postpartum PTSD symptoms and depression on the couple relationship and the parent-baby bond with a convenience sample, one study found that postpartum PTSD had no direct effect on the couple relationship, but the effect of PTSD on the couple relationship was fully mediated by symptoms of postpartum depression (Parfitt and Ayers, 2009). Research is needed to provide a more detailed account of how factors such as depressive symptomatology may mediate the association between PTSD symptoms and couple relationship satisfaction.

When examining the association between postpartum PTSD and couple satisfaction, potential third variables that may be related to both postpartum PTSD and couple satisfaction have to be taken into account. For example, couple relationship satisfaction is associated with maternal sociodemographic factors, such as age (Hershkowitz et al., 2017) and educational background (Blum and Mehrabian, 1999). These maternal sociodemographic factors as well as prior PTSD have been shown to also be risk factors for postpartum PTSD in community samples (Grekin and O’Hara, 2014; Tabet et al., 2016). Negative life events and mental health problems prior to birth (and PTSD in particular) are strong risk factors for postpartum PTSD, as well as for relationship dissatisfaction (Grekin and O’Hara, 2014; Lian et al., 2014; Tabet et al., 2016). Regarding infant factors, temperament is related to maternal sensitivity and parenting, both of which are related to the couple relationship (Lee, 2013). A difficult infant temperament has also been shown to be related to maternal PTSD symptoms after birth (Garthus-Niegel et al., 2017). Thus, the present study includes maternal age, educational background, negative life events, and infant temperament as covariates in the analyses to control for the potential confounding effects of these variables.

An international group of researchers and clinicians from the UK and other European countries stated the need for quantitative research using prospective studies with large, representative samples to investigate the possible negative impact of postpartum PTSD on relationships (McKenzie-McHarg et al., 2015). Therefore, this study that draws on data examining various risk factors and consequences of postpartum PTSD (Garthus-Niegel et al., 2013, 2014a,b, 2015, 2017) aimed to investigate the prospective association between maternal postpartum PTSD symptoms and couple relationship satisfaction 2 years later while controlling for important background variables. In particular, we aimed to test the following hypotheses:

Hypothesis 1: We hypothesized that postpartum PTSD will be prospectively associated with low couple relationship satisfaction, even when taking into account a variety of potential confounding variables.
Hypothesis 2: We hypothesized that the effect of postpartum PTSD symptoms on couple relationship satisfaction will be mediated by postpartum depression symptoms.

## Materials and Methods

### Study Population

The Akershus Birth Cohort study is a prospective cohort study which targeted all women scheduled to give birth at Akershus University Hospital, Norway. Recruitment took place from November 2008 to April 2010. Women were recruited for the study during their routine fetal ultrasound examination, which is performed at 17 weeks gestation, and were asked to complete questionnaires at 17 weeks gestation, 32 weeks gestation, 8 weeks postpartum, and 2 years postpartum. Of the eligible women, 80% (*n* = 3,752) agreed to participate and returned the first questionnaire. The number of eligible women dropped somewhat during the study time because some women had moved or were withdrawn from the study due to severe birth complications. Response rates were 81% (2,936 out of 3,621), 79% (2,217 out of 2,806), and 73% (1,480 out of 2,019), respectively. Detailed information regarding participation and drop out in the study has been published elsewhere (Garthus-Niegel et al., 2015, 2017).

For the present study, we used questionnaire data from pregnancy week 17 and 32, 8 weeks, and 2 years postpartum as well as data obtained from the hospital’s birth record. Data from the birth record were electronically recorded by hospital staff, including socio-demographic and medical information about the woman, the delivery, and the child.

The Akershus Birth Cohort study obtained ethical approval from the Regional Committees for Medical and Health Research Ethics (approval number S-08013a), and all participants provided written informed consent.

### Measures

#### Couple Relationship Satisfaction

Two years postpartum, couple relationship satisfaction was measured using the Relationship Satisfaction (RS10) Scale. The scale is a modified and shortened version of Mehrabian’s Marital Satisfaction Scale used in previous Norwegian studies (Røsand et al., 2011, 2012). The RS10 scale has good psychometric properties, high structural and predictive validity, and correlates 0.92 with The Quality of Marriage Index (Norton, 1983; Røsand et al., 2011, 2012). The scale contains 10 items, such as “I am satisfied with the relationship with my partner” and “My husband/partner and I have a close relationship.” The response categories ranged from 1 (strongly disagree) to 4 (strongly agree). The sum score ranges from 10 to 40 and higher scores reflect a larger degree of couple relationship satisfaction. Reliability in the current sample was excellent (α = 0.93).

#### Postpartum PTSD Symptoms

The Impact of Event Scale (IES) (Horowitz et al., 1979) was used to measure birth related PTSD symptoms at 8 weeks postpartum. The instrument is a self-rating scale that measures symptoms of intrusion (seven items) and avoidance (eight items). The scale has four response categories with the following weightings: 0 = not at all, 1 = rarely, 3 = sometimes, and 5 = often. Sum scores of the overall scale were computed (range 0–75), where higher scores reflect a higher degree of post-traumatic stress. Participants were instructed to complete the scale in relation to their childbirth. The IES has been previously validated in postpartum women (Olde et al., 2006) and can be used as a continuous or categorical measure, with scores more than 19 reflecting clinically significant distress, and scores more than 34 indicating that PTSD is likely to be present (Neal et al., 1994). Reliability in the present study was good (α = 0.84).

#### Postpartum Depression Symptoms

Symptoms of depression during the past week were measured using the Edinburgh Postnatal Depression Scale (EPDS) (Cox et al., 1987) at 8 weeks postpartum. The EPDS is a 10-item self-rating scale designed to identify postnatal depression. However, it is comprized of distinct and correlated depression and anxiety subscales (Ross et al., 2003; Jomeen and Martin, 2005). The scale has four response categories ranging from 0 to 3; thus, the total scores can range from 0 to 30. Higher scores reflect higher levels of depression, and a score ≥12 is indicative of a depressive disorder (Cox et al., 1987, 1996). Reliability in our sample was good (α = 0.85).

#### Background Variables

Information on parity (nulliparous “0” versus parous “1”) was assessed during pregnancy week 17. Further, age at delivery and maternal education were obtained from the hospital birth records. Educational level was coded as “0” (≤12 years of education) and “1” (>12 years of education). Paid employment was assessed 2 years postpartum by women’s self-report. According to Norwegian definitions (Haland and Glad, 2005), employment was defined as: (0) no paid employment, (1) part-time employment (between 1 and 36 h/week), and (2) full-time employment (≥37 h/week).

Regarding PTSD symptoms prior to birth, the women in our study reported at pregnancy week 17 whether at any time in their life they had been involved in or had experienced a dramatic or terrifying event. If this was the case, they reported whether they had suffered from eight potential symptoms related to that event during the last month. The symptoms were based on questions included in the Mini-International Neuropsychiatric Interview, which is designed for epidemiological studies and clinical trials. The Mini-International Neuropsychiatric Interview is a short structured clinical interview which enables researchers to make diagnoses of psychiatric disorders according to DSM-IV or ICD-10 (Sheehan et al., 1998). We measured symptoms as follows: “During the last month I… (1) “re-experienced the event (e.g., in dreams, nightmares, intense memories, or flashbacks),” (2) “avoided thinking or talking about the event,” (3) “had problems remembering the event,” (4) “felt distant,” (5) “had problems sleeping,” (6) “had problems concentrating,” (7) “have been nervous,” and (8) “ have been considerably disturbed by the event in my work and in social activities.” Depending on whether the symptom was present, a score was given or not. This resulted in a symptom score ranging from 0 (no symptoms) to 8 (maximum number of symptoms).

Adverse life events during the last 12 months were measured at pregnancy week 32 by seven selected items from existing life event scales (Coddington, 1972; Swearingen and Cohen, 1985). The following life events were included: (1) problems or conflicts with family members, friends, or neighbors, (2) problems at work or at the place of education, (3) economic problems, (4) serious illness or injury, (5) serious illness or injury in the close family, (6) traffic accident, fire, or theft, and (7) loss of a close relative. The answers were scored according to the woman’s reported grade of severity (emotionally not that difficult “1,” difficult “2,” very difficult “3”), and total scores can range from 0 to 21.

At 8 weeks postpartum, infant temperament was measured with a 10-item adapted version of the “Fussy/Difficult” subscale of the Infant Characteristics Questionnaire (Bates et al., 1979). This scale assesses infant difficultness as perceived by the primary caregiver. Women rated their infants’ usual mood and temperament on a seven-point rating scale, with higher scores reflecting greater infant difficultness. Reliability was good (α = 0.82).

### Statistical Analyses

Analyses were conducted in the framework of structural equation modeling (Bollen, 1989), and we used the statistical program Mplus 8 (Muthén and Muthén, 1998–2017) for all analyses. A robust weighted least squares estimator (WLSMV) was employed because some of the items included in the analyses (i.e., items from the IES and the EPDS) were considered to be ordered categorical variables (Muthén and Muthén, 1998–2017).

First, we conducted a confirmatory factor analysis of the RS10 Scale, constructing a latent factor for couple relationship satisfaction. Likewise, a latent factor for postpartum PTSD was modeled by means of confirmatory factor analysis. Further, for the EPDS a second order confirmatory factor analysis was conducted, as the EPDS has been shown to have a bi-dimensional factor structure with a depression and an anxiety component (Ross et al., 2003; Jomeen and Martin, 2005). For this purpose, we first constructed two latent factors loading on the depression and anxiety items of the scale, respectively (“depression factor”: EPDS items 1, 2, and 6–10, “anxiety factor”: EPDS items 3–5). In addition, an overall EPDS factor was constructed, based on the two lower order latent factors.

As conducting confirmatory factor analyses for all scales would have led to an excessively complex model, we chose to construct latent variables only for the most important psychological variables, i.e., the main predictor, the mediator, and the outcome. The remaining background variables were treated as manifest variables. Correlation analyses were conducted to study the bivariate associations among all included variables. Further, all latent and manifest variables that were significantly related to couple relationship satisfaction were entered into a multivariate mediation model. Finally, in order to differentiate the distinct relationship of the depression and the anxiety component of the EPDS, we also estimated a multivariate mediation model with those two components separately, each being represented by their own latent factor.

We conducted mediation analyses to test whether or not the indirect effects involving the putative mediators were statistically significant (Hayes, 2009). More specifically, standard errors of the mediation effects were estimated by the product of coefficients approach in a path analytic framework (Hayes, 2009). As recommended in the literature (Hayes, 2009), we estimated bias corrected standard errors of the mediation effects by means of bootstrapping based on 5,000 bootstrap samples.

As a considerable number of participants had dropped out during the course of the study (Garthus-Niegel et al., 2015, 2017), we performed attrition analyses. More specifically, we included relevant socio-demographic and mental health variables (i.e., maternal age, education, symptoms of depression and anxiety, and prior PTSD symptoms) assessed at pregnancy week 17 and the hospital birth record simultaneously as predictors of drop-out within 2 years postpartum in multiple logistic regression analyses. The results showed that women with higher education (OR.57, 95% CI 0.49–0.66, *p* < 0.001) and older age (OR.97, 95% CI 0.95–0.98, *p* < 0.001) were less likely to drop out of the study. Conversely, women with a high symptom load of depression (OR 1.05, 95% CI 1.02–1.07, *p* < 0.001) were somewhat more likely to drop out. Symptoms of anxiety and PTSD were not significantly related to drop-out (*p* > 0.05).

Missing data were accounted for by the missing routines for WLSMV in Mplus, which are based on pairwise present analysis (Muthén and Muthén, 1998–2017). Our final sample consisted of 2,106 women.

## Results

**Table 1** shows descriptive statistics for all variables. Mean maternal age at birth was 31.33 (*SD* = 4.60) years, 67.6% of women had an educational level beyond high school, and 49.1% reported this pregnancy to be their first one.

**Table 1:** Means and SDs of all study variables.

Measures	*N* (%) or Mean ± SD
Couple relationship satisfaction	33.59 ± 5.47
Postpartum PTSD symptoms	7.00 ± 8.25
Postpartum depression symptoms	4.47 ± 4.08
**Background variables**	
Parity	
Primiparous	1,033 (49.1)
Multiparous	1,073 (50.9)
Age	31.33 ± 4.60
**Educational level**	
>12 years	1,424 (67.6)
≤12 years	682 (32.4)
**Paid employment**	
Full-time employment	763 (61.5)
Part-time employment	435 (35.1)
No employment	43 (3.4)
Prior PTSD symptoms	0.25 ± 0.76
Negative life events	1.27 ± 2.22
Difficult infant temperament	25.70 ± 9.02

At 8 weeks postpartum, 2.0% of all participating women had probable postpartum PTSD (scores above 34). The mean IES score was 7.00 (*SD* = 8.25). Prevalence of likely depression was 7.0%, with a mean EPDS score of 4.47 (*SD* = 4.08). Thus, results indicated low symptom burden for both postpartum PTSD and depression symptoms, as expected in a community sample such as the present one.

Two years postpartum, the vast majority of women (95.9%) were married or living with a partner, and couple relationship satisfaction was relatively high, with a mean score of 33.59 (*SD* = 5.47), thereby indicating that women on average would agree to positive descriptions of their couple relationship.

The confirmatory factor analysis of the RS10 Scale showed an acceptable fit for a one factor solution (root mean square error of approximation (RMSEA) = 0.074, comparative fit index (CFI) = 0.97, Tucker–Lewis index (TLI) = 0.96). Similarly, model fit indices indicated an acceptable fit for the one factor model for the IES (RMSEA = 0.064, CFI = 0.95, TLI = 0.95) and a second order model for the EPDS (RMSEA = 0.078, CFI = 0.98, TLI = 0.97).

**Table 2** shows intercorrelations among all study variables. As expected, most background variables significantly negatively predicted couple relationship satisfaction. In addition, postpartum PTSD symptoms (*r* = −0.16, *p* < 0.001) and depression symptoms (*r* = −0.29, *p* < 0.001) were negatively related to subsequent couple relationship satisfaction.

**Table 2:** Intercorrelations between all included study variables.

	(1)	(2)	(3)	(4)	(5)	(6)	(7)	(8)	(9)
(1) Couple relationship satisfaction									
(2) Postpartum PTSD symptoms	−0.16^∗∗∗^								
(3) Postpartum depression symptoms	−0.29^∗∗∗^	0.53^∗∗∗^							
(4) Parity	−0.06^∗^	−0.17^∗∗∗^	−0.04						
(5) Age	−0.09^∗∗∗^	−0.13^∗∗∗^	−0.08^∗∗^	0.33^∗∗∗^					
(6) Educational level	0.07^∗∗^	−0.07^∗∗^	−0.06^∗^	0.07^∗∗∗^	0.33^∗∗∗^				
(7) Paid employment	0.03	0.02	−0.01	−0.04	0.06^∗∗^	0.07^∗∗^			
(8) Prior PTSD symptoms	−0.09^∗∗∗^	0.18^∗∗∗^	0.26^∗∗∗^	−0.03^∗^	−0.15^∗∗∗^	−0.13^∗∗∗^	−0.05^∗^		
(9) Negative life events	−0.11^∗∗∗^	0.15^∗∗∗^	0.28^∗∗∗^	0.05^∗∗^	−0.06^∗∗∗^	−0.06^∗∗∗^	−0.04	0.16^∗∗∗^	
(10) Difficult infant temperament	−0.14^∗∗∗^	0.19^∗∗∗^	0.36^∗∗∗^	−0.12^∗∗∗^	−0.07^∗∗^	0.01	−0.01	0.06^∗∗^	0.08^∗∗∗^

*^∗^p < 0.05, ^∗∗^p < 0.01, ^∗∗∗^p < 0.001.*

**Table 3**, Model 1, displays results from structural equation models where postpartum PTSD symptoms and all background variables except maternal paid employment were included as predictors of couple relationship satisfaction. In line with Hypothesis 1, results showed that postpartum PTSD was significantly associated with couple relationship satisfaction (β = −0.11, *p* < 0.01) even when controlling for covariates. Moreover, parity, age, prior PTSD symptoms, negative life events, and difficult infant temperament significantly predicted couple relationship satisfaction in the multiple model.

**Table 3:** Results from structural equation models predicting couple relationship satisfaction.

Predictors	Model 1, β	Model 2, β	Model 3, β
Postpartum PTSD symptoms	−0.11^∗∗^	−0.02	−0.07
Postpartum depression symptoms		−0.19^∗∗∗^	
Depression factor			−0.21^∗∗∗^
Anxiety factor			0.06
Background variables			
Parity	−0.07^∗^	−0.07^∗^	−0.07^∗^
Age	−0.13^∗∗∗^	−0.13^∗∗∗^	−0.13^∗∗∗^
Educational level	0.04	0.04	0.04
Prior PTSD symptoms	−0.08^∗∗^	−0.08^∗∗^	−0.08^∗∗^
Negative life events	−0.09^∗∗∗^	−0.09^∗∗∗^	−0.09^∗∗∗^
Difficult infant temperament	−0.15^∗∗∗^	−0.15^∗∗∗^	−0.15^∗∗∗^

*β = standardized regression coefficient, ^∗^p < 0.05, ^∗∗^p < 0.01, ^∗∗∗^p < 0.001.*

Next, further analyses were conducted by including postpartum depression symptoms as additional predictor in the model (see **Table 3**, Model 2). Results from these analyses showed postpartum depression symptoms to be significantly related to couple relationship satisfaction (β = −0.19, *p* < 0.001); however, postpartum PTSD symptoms as predictor diminished to non-significance when including depressive symptoms. When conducting additional analyses to differentiate the distinct influence of the depression and the anxiety component of depression symptoms, we only found the depression factor to be significantly negatively related to couple relationship satisfaction (β = −0.21, *p* < 0.001), whereas the anxiety component did not significantly predict the outcome (see **Table 3**, Model 3).

**FIGURE 1:**
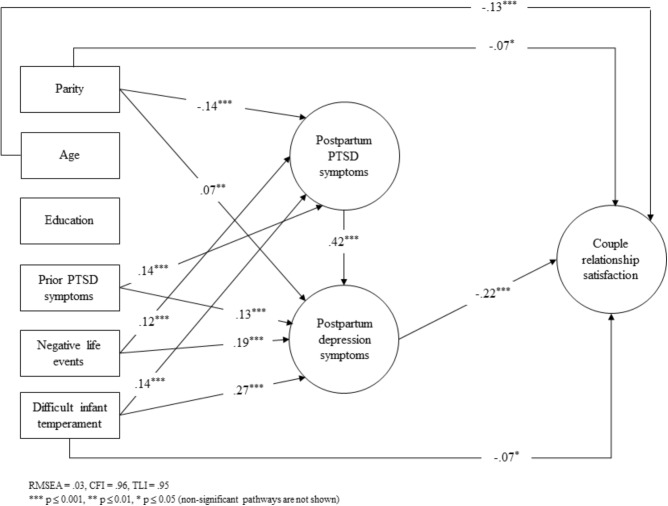
Full mediation model predicting couple relationship satisfaction. Abbreviations: CFI, comparative fit index; EPDS, Edinburgh Postnatal Depression Scale; IES, Impact of Event Scale; PTSD, posttraumatic stress disorder; RMSEA, root mean square error of approximation; RS10, Relationship Satisfaction Scale; TLI, Tucker–Lewis index.

The diminishing association between postpartum PTSD symptoms and couple relationship satisfaction in Models 2 and 3 indicates that postpartum depression symptoms may act as a mediator. A final set of analyses were thus conducted to formally test for mediation by using bootstrapping. More specifically, the structural equation model as depicted in **Figure 1** was specified. Only the statistically significant pathways are shown. The model showed a good fit (RMSEA = 0.027, CFI = 0.96, TLI = 0.95) and was therefore deemed to be an appropriate model.

In line with Hypothesis 2, tests of indirect effects showed that the negative effect of postpartum PTSD symptoms on couple relationship satisfaction was fully mediated by depression symptoms (standardized indirect effect through depression symptoms = −0.09, 95% CI: −0.13; −0.06). Moreover, the association between postpartum PTSD symptoms and couple relationship satisfaction was no longer significant.

## Discussion

This study investigated the prospective association between maternal postpartum PTSD symptoms and couples’ relationship satisfaction 2 years postpartum while controlling for important background variables. Postpartum PTSD symptoms were prospectively related to low couple relationship satisfaction at 2 years postpartum, even when controlling for a considerable number of background factors. When including postpartum depression symptoms as predictor in the analyses, the effect of postpartum PTSD was no longer significant. Moreover, more detailed analyses showed that postpartum depression symptoms acted as a significant mediator, fully explaining the association of postpartum PTSD with couples’ relationship satisfaction. Differentiating the distinct influence of depression and anxiety symptoms, we found that it was the depressive and not the anxiety component that predicted low couple relationship satisfaction at 2 years postpartum.

Our results of a prospective association between postpartum PTSD symptoms and couple relationship satisfaction are consistent with previous studies in war veterans and civil samples (Gewirtz et al., 2010; Taft et al., 2011; Campbell and Renshaw, 2013; Hershkowitz et al., 2017). They also resonate with qualitative findings in women following traumatic childbirth that describe “shattered relationships” and perceived lack of communication and support (Nicholls and Ayers, 2007; Fenech and Thomson, 2014). Previous studies are limited by a low response rate, small sized samples, and short follow-up periods. Our large-scale prospective study with an acceptable response rate and a long follow-up (2 years) therefore makes an important contribution to the literature.

The role of depression symptoms as a mediator in our study is in agreement with results from one previous study showing that the effect of postpartum PTSD on the couple relationship was mediated by depression (Parfitt and Ayers, 2009). This is not surprising, given the high comorbidity of postpartum PTSD with postpartum depression (Czarnocka and Slade, 2000; Grekin and O’Hara, 2014). Furthermore, postpartum depression has previously been shown to be associated with poorer postpartum marital relationship satisfaction (Wenzel et al., 2005) and perceived couple relationship quality (Malus et al., 2016). Negative cognitions typical for depression are likely to affect the perception of the couple relationship. This was illustrated in a qualitative study showing that some women after traumatic childbirth felt they were no longer worthy of the relationship (Fenech and Thomson, 2014). Our population-based study provides a detailed account of how depressive symptomatology mediates the association between PTSD symptoms and couple relationship satisfaction and thus contributes to a better understanding of this important problem.

Our model showed that postpartum PTSD symptoms predict depression symptoms, which in turn predict poor couple relationship satisfaction. Based on clinical observations, it has previously been suggested that postpartum depression is usually secondary to postpartum PTSD (McKenzie-McHarg et al., 2015). Therefore, PTSD continues to have a significant indirect effect on couple relationship satisfaction. Finally, our results are strengthened by the fact that the depressive and not the anxiety component predicted low couple relationship satisfaction at 2 years postpartum.

Our findings have important clinical implications. Couple relationship quality is related to parenting and the attachment relationship the child forms with its parents (Coln et al., 2013; Hosokawa and Katsura, 2017). This, in turn is linked to the mental health and development of the child (Sroufe, 2005; Coln et al., 2013; Hosokawa and Katsura, 2017). The focus on parenting as a possible explanation for the association between the quality of the couple relationship and child outcomes stems from the “spillover hypothesis” stating that the affect and mood generated in the couple transfers to the parent-child relationship (Erel and Burman, 1995). The breakdown of the parental relationship has been shown to be related to social, emotional, behavioral, physical, and health problems, as well as poor educational achievement of the child, and socio-economic disadvantage (Coleman and Glenn, 2010; Vaez et al., 2015; Hosokawa and Katsura, 2017). Detecting couple relationship problems early and providing appropriate professional help may thus improve parenting and promote positive child outcomes. Furthermore, early identification of women with symptoms of postpartum PTSD or depression and rapid access to evidence-based treatment where appropriate are vital (National Institute for Health and Care Excellence [Nice], 2014).

Limitations of the study include the measurement of couple relationship satisfaction. We only measured the woman’s perception of the couple relationship, which is likely to be influenced by symptoms of psychopathology and other external risk factors. Furthermore, we did not assess the partner’s PTSD symptoms. The effect of PTSD on the couple relationship may differ depending on whether one or both partners experience PTSD. Some authors have argued that “the non-traumatized partner may have limited empathy and understanding for his or her partner” (p. 62) (Nelson et al., 2002). If both partners suffer from PTSD, then the mutual understanding and empathy might be increased, but “a mutual impact of the partners’ individual symptoms on one another” may persist (p. 60) (Nelson et al., 2002). The recently published Dyadic Responses to Trauma Model illustrates different ways in which a traumatic event can impact on the couple relationship (Marshall and Kuijer, 2017). It provides a possible framework to explain why some partners can be resilient or at risk for individual psychological reactions to trauma but also to relationship outcomes, and also depicts how these processes unfold dyadically (Marshall and Kuijer, 2017). It underlines previous findings that PTSD symptoms of each member of a couple were independently related to distress in their relationship (Riggs, 2014). Future studies should thus aim to prospectively measure couple relationship satisfaction and PTSD symptoms in both partners. Another limitation is the fact that postpartum PTSD and depression symptoms were measured at the same time. It is therefore not possible to provide definite answers about their temporal relationship, i.e., which symptoms developed first. Also, we did not assess whether participants received medical treatment or psychotherapy during the first 2 years postpartum. Thus, we could not examine whether treatment might be an additional confounder of the association of postpartum PTSD and depression with couple relationship satisfaction.

Further, our sample was relatively homogeneous, mainly Caucasian. As we have shown previously, there is reason to believe that there is a slight social gradient associated with participation in the study (Garthus-Niegel et al., 2014b, 2015). Likewise, there was somewhat selective attrition during the longitudinal course of the study, as demonstrated by attrition analyses. However, it is important to bear in mind that selection bias does not necessarily influence the results when associations between variables are investigated (Nilsen et al., 2009).

## Conclusion

Our model showed that PTSD symptoms predict depression symptoms, which in turn predict poor couple relationship satisfaction. PTSD, therefore, continues to have a substantial indirect effect on couple relationship satisfaction. Differentiating the distinct influence of the depression and the anxiety symptoms, the results showed that it was the depressive and not the anxiety component that predicted low couple relationship satisfaction at 2 years postpartum. Early detection of couples’ relationship problems and the provision of professional help, particularly in high-risk couples—where at least one partner shows maladaptive coping or develops mental health symptoms, such as PTSD or depression following childbirth—may not only improve the quality of the couple relationship but also improve parenting and promote positive child outcomes.

## Ethics Statement

This study was carried out in accordance with the recommendations of the Norwegian Committee for Ethics in Medical Research. All subjects gave written informed consent in accordance with the Declaration of Helsinki. The protocol was approved by the ABC steering group at the hospital and by the Regional Research and Ethic Committee in South East Norway (approval number S-08013a).

## Author Contributions

SG-N, AH, TvS, and EH contributed to the conception and design of the study. AH and EH did literature search. SG-N and TvS performed the statistical analysis. SG-N and AH wrote the first draft of the manuscript. SA contributed with her expertise in the research field. KW contributed with her clinical expertise. ME-G designed the data collection instruments, coordinated, and supervised data collection. All authors contributed to manuscript revision, read, and approved the submitted version.

## Conflict of Interest Statement

The authors declare that the research was conducted in the absence of any commercial or financial relationships that could be construed as a potential conflict of interest.
